# Neurite outgrowth stimulatory effects of culinary-medicinal mushrooms and their toxicity assessment using differentiating Neuro-2a and embryonic fibroblast BALB/3T3

**DOI:** 10.1186/1472-6882-13-261

**Published:** 2013-10-11

**Authors:** Chia-Wei Phan, Pamela David, Murali Naidu, Kah-Hui Wong, Vikineswary Sabaratnam

**Affiliations:** 1 Mushroom Research Centre, Institute of Biological Sciences, Faculty of Science, University of Malaya, 50603 Kuala Lumpur, Malaysia; 2 Institute of Biological Sciences, Faculty of Science, University of Malaya, 50603 Kuala Lumpur, Malaysia; 3 Department of Anatomy, Faculty of Medicine, University of Malaya, 50603 Kuala Lumpur, Malaysia

**Keywords:** Culinary-medicinal mushrooms, Neurite outgrowth, Cytotoxicity, Mouse neuroblastoma N2a cell, Mouse 3T3 embryonic fibroblast, Neurofilament

## Abstract

**Background:**

Mushrooms are not only regarded as gourmet cuisine but also as therapeutic agent to promote cognition health. However, little toxicological information is available regarding their safety. Therefore, the aim of this study was to screen selected ethno-pharmacologically important mushrooms for stimulatory effects on neurite outgrowth and to test for any cytotoxicity.

**Methods:**

The stimulatory effect of mushrooms on neurite outgrowth was assessed in differentiating mouse neuroblastoma (N2a) cells. Neurite length was measured using Image-Pro Insight processor system. Neuritogenesis activity was further validated by fluorescence immunocytochemical staining of neurofilaments. *In vitro* cytotoxicity was investigated by using mouse embryonic fibroblast (BALB/3T3) and N2a cells for any embryo- and neuro-toxic effects; respectively.

**Results:**

Aqueous extracts of *Ganoderma lucidum*, *Lignosus rhinocerotis*, *Pleurotus giganteus* and *Grifola frondosa*; as well as an ethanol extract of *Cordyceps militaris* significantly (p < 0.05) promoted the neurite outgrowth in N2a cells by 38.4 ± 4.2%, 38.1 ± 2.6%, 33.4 ± 4.6%, 33.7 ± 1.5%, and 35.8 ± 3.4%; respectively. The IC_50_ values obtained from tetrazolium (MTT), neutral red uptake (NRU) and lactate dehydrogenase (LDH) release assays showed no toxic effects following 24 h exposure of N2a and 3T3 cells to mushroom extracts.

**Conclusion:**

Our results indicate that *G. lucidum*, *L. rhinocerotis*, *P. giganteus*, *G. frondosa* and *C. militaris* may be developed as safe and healthy dietary supplements for brain and cognitive health.

## Background

Neurite outgrowth is an important event in neuronal path finding and the establishment of synaptic connections during development [1,2]. It is also essential in neuronal plasticity, neuronal regeneration after injury [3,4] and neurodegenerative conditions such as Alzheimer’s and Parkinson’s diseases [5]. Therefore, treatments aiming at promoting neurite outgrowth and preserving the neurite network and synaptic connections are needed.

The potential use of culinary-medicinal mushrooms in neurodegenerative diseases is being explored [6]. On-going research in our laboratory shows that *Hericium erinaceus* (Bull.: Fr) Pers. (monkey’s head mushroom, lion’s mane mushroom and Yamabushitake) [7]*, Lignosus rhinocerotis* (Cooke) Ryvarden (tiger milk mushroom) [8,9], and *Pleurotus giganteus* (Berk.) Karunarathna & K.D. Hyde (morning glory mushroom, cow’s stomach mushroom) [10] exhibit neurite outgrowth stimulatory effects in NG108-15 and PC12 cell lines. This observation raised a question with respect to the neurodevelopmental effects, if any, of culinary-medicinal mushrooms. Birth defects have been identified as a growing social and healthcare issue. Congenital diseases are present in 2–3% of human newborns [11]. About 20% of the birth defects are due to genetic anomaly and 10% are caused by environmental factors during pregnancy [12]. Therefore, toxicological safety assessments of food, chemicals and drugs to evaluate the effects on reproductive health and for embryotoxicity have become an important requirement. Thus, the aims of the present study were (a) to evaluate neurite outgrowth stimulatory effects of selected culinary-medicinal mushrooms using neuroblastoma-2a (N2a) cells and (b) to assess the neuro- and embryotoxicity of the mushroom extracts using N2a and 3T3 fibroblasts. The results will enable us to select potential mushrooms for further in depth *in vivo* developmental toxicity evaluation.

## Methods

### Mushroom and plant samples

The mushrooms were authenticated by experts in the Mushroom Research Centre, University of Malaya and voucher specimens were deposited in the University of Malaya herbarium at Rimba Ilmu (Table 1). Fresh fruiting bodies of *Ganoderma lucidum* (Fr) P. Karst (KLU-M 1233) and *H. erinaceus* (KLU-M 1232) were obtained from Ganofarm Sdn Bhd. *Pleurotus giganteus* (KLU-M 1227) was provided by Nas Agro Farm and Dong Foong Biotech. Freeze dried powder of *Cordyceps militaris* (L.:Fr.) Link and *L. rhinocerotis* were purchased from BioFact Life Sdn Bhd and Ligno Biotek Sdn Bhd, respectively. *Pleurotus pulmonarius* (Fr.) Quél. (KLU-M 1309) and *Gingko biloba* extracts were obtained from Reishilab Sdn Bhd, Selangor. Wild *Ganoderma neo-japonicum* Imazeki 1939 (KLU-M 1231) was collected from forest in Ulu Grik, Perak, Malaysia. *Ganoderma neo-japonicum* was used as traditional medicine by the indigenous people in Malaysia. *Grifola frondosa* (Dicks.: Fr.) S.F. Gray (KLU-M 1229) imported from Japan was obtained from supermarkets in Selangor, Malaysia. *Lycium barbarum (*wolfberry), a traditional Chinese medicine was purchased from a Chinese medicine shop in Selangor, Malaysia and deposited in University of Malaya herbarium. Curcumin was purchased from NatXtra, Synthite Co., India.

**Table 1:** Medicinal mushrooms used in this study, their common names, and culinary nature

**Mushroom species**	**Voucher number**	**Common names**	**Local names (in Malay)**	**Part used**	**Edible/**	**Wild/**	**Medicinal properties**	**References**
					**culinary**	**cultivated**		
*Pleurotus giganteus*	KLU -M 1227	Zhudugu, cow’s stomach mushroom	*Cendawan seri pagi* (morning glory)*, perut lembu* (cow’s stomach)	Fruiting body	Culinary	Cultivated	Antioxidant, neurite outgrowth simulation	[10,13]
*Pleurotus pulmonarius*	KLU-M 1309	Grey oyster mushroom	*Cendawan tiram kelabu* (grey oyster)	Fruiting body	Culinary	Cultivated	Antioxidant, anti-diabetic	[14]
*Lignosus rhinocerotis*	Purchased from Ligno Biotek Sdn Bhd	Tiger milk mushroom,	*Cendawan susu rimau*	Sclerotium and mycelium	Non-culinary but edible	Cultivated	Anticancer, anti-inflammatory, neurite outgrowth stimulation	[8,15]
			(tiger’s milk)					
*Hericium erinaceus*	KLU-M 1232	Monkey’s head mushroom, lion’s mane mushroom, Yamabushitake	*Cendawan bunga kubis*	Fruiting body	Culinary	Cultivated	Anti-ulcer, neurite outgrowth stimulation	[7,16-18]
			(cauliflower)					
*Ganoderma lucidum*	KLU-M 1233	Lingzhi, reishi	*Cendawan merah* (red mushroom)	Fruiting body	Non-culinary but edible	Cultivated	Anticancer, neuroprotection	[19]
*Ganoderma neo-japonicum*	KLU-M 1231	Purple reishi	*Cendawan senduk* (cobra mushroom)	Fruiting body	Non-culinary but edible	Wild	Antioxidant, antihepatoxic, neurite outgrowth stimulation	[9,20]
*Cordyceps militaris*	Purchased from BioFact Life Sdn Bhd	Winter worm summer grass, caterpillar fungus	-	Fruiting body (ascocarp)	Non-culinary but edible	Cultivated	Anti-inflammatory, anticancer, relief respiratory disorders	[21]
*Grifola frondosa*	KLU- M 1229	Maitake, hen of the woods	*Cendawan maitake*	Fruiting body	Culinary	Cultivated	Anti-inflammatory, anti-cholesterol	[22]

### Chemicals

Phosphate buffered saline (PBS), [3-(4,5-dimethythiazol-2-yl)-2,5-diphenyltetrazolium bromide] (MTT), dimethyl sulfoxide (DMSO), nerve growth factor (NGF) from murine submaxillary gland, 3-amino-7-dimethylamino-2-methyl-phenazine hydrochloride (neutral red), and foetal bovine serum (FBS) were obtained from Sigma Co. (St. Louis, MO, USA).

### Cell culture

Mouse neuroblastoma cells (N2a, ATCC CCL-131) and mouse embryonic fibroblast cells (BALB/c 3T3, ATCC clone A31) were purchased from American Type Culture Collection (ATCC; MD, USA). N2a cells were cultured in Eagle’s minimum essential medium (MEM) with L-glutamine (PAA) supplemented with 10% (v/v) heat-inactivated foetal bovine serum (PAA), 100 U/ml penicillin, and 100 μg/ml streptomycin. 3T3 fibroblasts were maintained in Dulbecco’s modified Eagle’s medium (DMEM) with L-glutamine (high glucose at 4.5 g/l) supplemented with 10% FBS. All the cells were maintained at 37°C and 5% CO_2_ in a humidified atmosphere. N2a cells were subcultured at 3 – 4 days intervals while 3T3 cells were routinely passaged every 2 – 3 days. For preservation, the cells were frozen at -70°C liquid nitrogen in complete medium supplemented with 5% (v/v) dimethyl sulfoxide (DMSO; Sigma) as a cryoprotecting agent.

### Preparation of mushroom extracts

The fresh fruiting bodies of *P. giganteus*, *P. pulmonarius*, *H. erinaceus*, and *G. frondosa* were sliced, frozen and then freeze-dried for two days. The freeze-dried fruiting bodies were then ground to powder and kept at 4 - 8°C. For aqueous extraction, the freeze dried powder was soaked in distilled water (1:20, w/v) at room temperature and 200 rpm in a shaker for 24 h. The mixture was then double boiled in water bath for 30 min, cooled and then filtered (Whatman No. 4). The resulting aqueous extracts were freeze-dried and kept at -20°C prior to use. The process was repeated for the freeze-dried powder of *C. militaris* and *L. rhinocerotis* sclerotia. For ethanol extraction, the freeze dried powder was soaked in 95% ethanol at room temperature for three days and the process was repeated three times. The solvent was then evaporated using a rotary evaporator (Eyela N-1000, USA) to give a brownish viscous extract.

### Neurite outgrowth assay

N2a cells were seeded in 24-well culture plate at an initial density of 5,000 cells per well containing complete growth medium (1 ml/well) and incubated overnight. Concentrations of NGF ranging from 5–100 ng/ml (w/v) were tested to determine the optimum concentration that stimulates maximum neurite outgrowth. The optimum concentration was then used as a positive control throughout the subsequent assays. Aqueous and ethanol mushroom extracts were stocked at 10 mg/ml and were subsequently dissolved in sterile distilled water or DMSO, to the appropriate concentrations. The final concentration of DMSO in the assays was 0.1 - 0.25%. To induce cell differentiation, the complete medium was carefully replaced with 5% serum medium before exposure to mushroom extracts at 10–50 μg/ml. Cells with medium only served as a negative control. All the cells were incubated for 48 h at 37°C, 95% air and 5% CO_2_ to observe neuritogenesis activity, if any. Curcumin, *G. biloba* and *L. barbarum* extracts were also tested to compare the neurite outgrowth activities with those of mushroom extracts.

### Quantification of neurite bearing cells

Five random fields (100 – 200 cells/well) were examined in each well by using a phase contrast microscope (20× magnifications) equipped with QImaging Go-3 camera (QImaging, Canada). Neurite length was measured in at least 30 cells in randomly chosen fields by using image processor system Image-Pro Insight (MediaCybernetics, MD). The number of neurite outgrowths, defined as axon-like extensions that were double or more than the length of the cell body diameter was recorded. The percentage of neurite bearing cells (%) is the number of neurite bearing cells divided by the total number of cells in a field and then multiplied by 100%. At least three independent experiments were conducted and results were expressed as mean ± standard deviation (S.D).

### Fluorescence immunocytochemistry study

The axon-like extensions were confirmed as neurite outgrowth by immunofluorescence study. N2a cells were seeded in 12-well μ-dishes (ibidi, Martinsried, Germany) and were exposed to treatments for 48 h. The cells were fixed with 4% paraformaldehyde in PBS (pH 7) for 20 min. After two washes with PBS, the cells were incubated with rabbit anti-neurofilament 200 polyclonal antibody (1:80 in 10% sheep serum as blocking buffer) for 1 h. The cells were washed and then incubated in a mixture of fluorescein isothiocyanate (FITC)-conjugated secondary antibody and sheep anti-rabbit IgG (1:160 in blocking buffer) for 2 h at room temperature in the dark. The cells were then washed three times. 4'-6-diamidino-2- phenylindole (DAPI) was used to counter stain the nuclei. Images were observed under a fluorescent microscope (Nikon Eclipse 80i microscope).

### Evaluation of embryo- and/or neurotoxic effects of mushroom extracts

#### MTT [3-(4,5-dimethylthiazol-2-yl)-2,5-diphenyltetrazolium bromide] assay

N2a and 3T3 cells (1 × 10^4^) per well were seeded in 96-well plates. After incubation for 24 h, different concentrations of mushroom extracts (0 – 5 mg/ml) dissolved in phenol red free culture medium were added to each well. The samples were incubated for 24 h at 37°C under humidified atmosphere of 5% CO_2_ and 20 μl MTT (5 mg/ml) was added to each well. The crystal dyes which were up-taken by the cells were then dissolved with DMSO. Absorbance was measured at 570 nm in a microplate reader (Tecan, Austria) using 630 nm as a reference wavelength. All measurements were done in triplicates, and at least three independent experiments were carried out. To calculate IC_50_ values which estimated the concentration of mushroom extract that caused 50% inhibition of proliferation (viability) in N2a and 3T3 cells, Probit analysis was conducted using SPSS 17.0 (SPSS Science Inc., Chicago, IL).

#### Neutral red uptake assay

Neutral red medium (40 μg/ml) was prepared fresh before use by diluting the neutral red stock (4 mg/ml) with phenol red free culture medium. Neutral red medium was centrifuged at 1800 rpm for 10 min to remove any precipitated dye crystals before use. After cell seeding and treatment (0 – 5 mg/ml), the medium was discarded and replaced with equal amount of neutral red medium to each well of the plate. The plate was then incubated for 2 h. The neutral red medium was then removed and the cells were washed quickly with adequate amount of PBS. A total of 100 μl of neutral red solubilising solution (1% acetic acid in 50% ethanol) was added to each well and allowed to stand for 10 minutes at room temperature until the neutral red extracted from the cells reached a homogeneous solution. The absorbance at a wavelength of 540 nm with 690 nm of background absorbance was spectrophotometrically measured (Tecan, Austria). The experiment was repeated at least three different times.

#### Lactate dehydrogenase (LDH) release assay

After cell seeding and treatment with mushroom extracts (0 – 5 mg/ml), the culture plates were centrifuged at 1500 rpm for 5 minutes and 50 μl of supernatant was then transferred to a new plate for LDH analysis according to manufacturer instruction (Sigma). To each well, 100 μl of LDH mixture solution comprising of LDH assay substrate, dye and cofactor was added and incubated at room temperature for 30 min. The reaction was stopped by adding 10 μl of 1 N HCl to each well. Absorbance was spectrophotometrically measured at 490 nm with background absorbance at 690 nm. Triton X-100 (0.5%, Scharlau) was used as a positive control and was thus set to 0% viability representing a 100% cell death.

### Statistical analysis

All the experimental data are expressed in mean ± standard deviation (S.D). Statistical differences between groups were analysed and calculated by one-way analysis of variance (ANOVA) from at least three independent experiments. This was followed by Duncan's multiple range tests. *P* < 0.05 was considered to be significant between groups.

## Results

### The effects of NGF on neurite outgrowth activity in N2a cells

**Figure 1:**
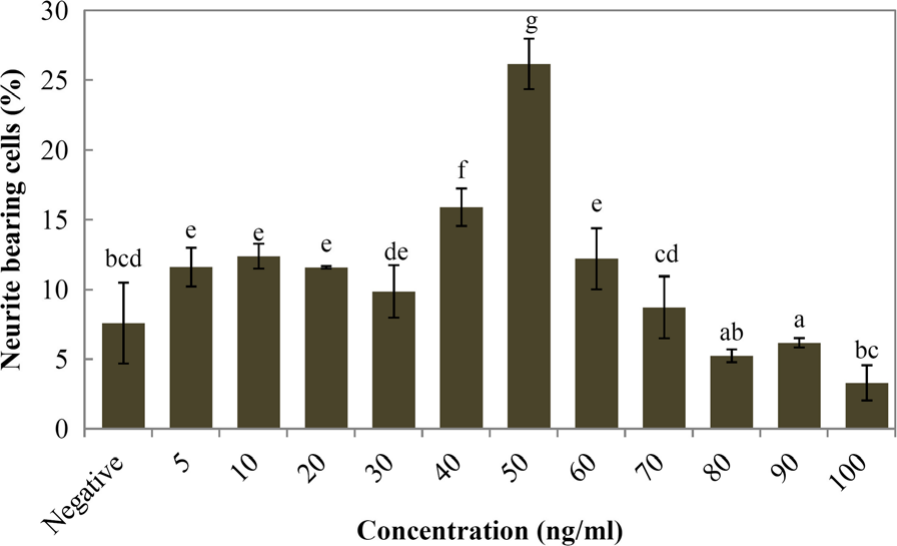
**Effects of different NGF concentrations (5 – 100 ng/ml) on stimulation of neurite outgrowth using differentiating N2a cells as an *in vitro *model.** The results shown represent the mean ± SD; n = 3. Means not sharing a common letter were significantly different at *p* < 0.05.

Nerve growth factor induced neurite outgrowth of N2a in a dose-dependent manner (Figure 1). After 48 h of NGF stimulation, the percentage of neurite bearing cells increased significantly (*p* < 0.05) to 26.1 ± 1.8% in N2a cells treated with 50 ng/ml NGF when compared to negative control (7.6 ± 2.5 %). At 60 ng/ml of NGF, the percentage of neurite bearing cells significantly decreased to 12.2 ± 2.1% (*p* < 0.05). Based on these findings, the optimised concentration of NGF (50 ng/ml) was selected for the following experiments as a positive control.

### The effects of different mushroom extracts on neurite outgrowth activity in N2a cells

**Figure 2:**
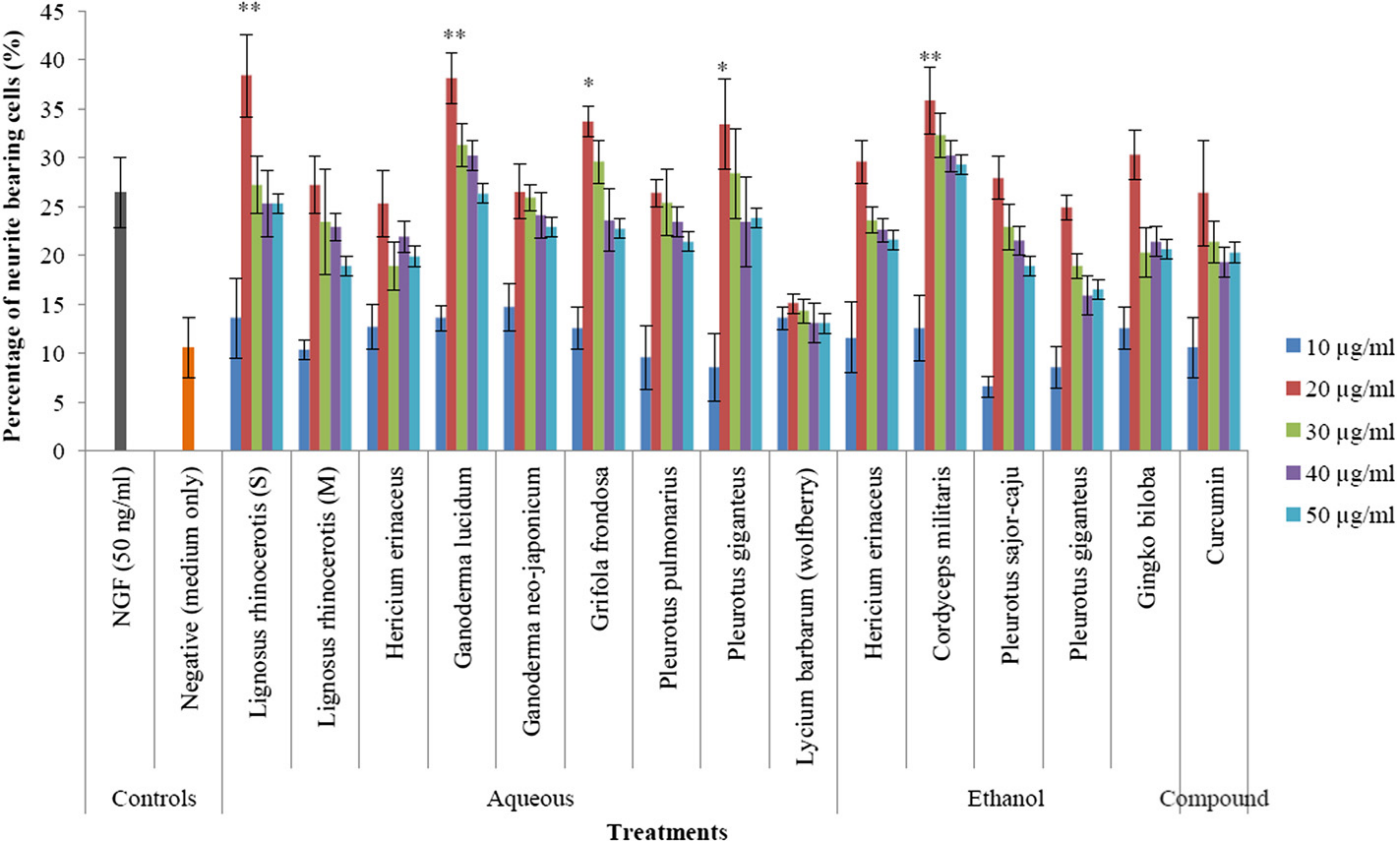
Percentage of neurite bearing cells after treatment of different mushroom and plant extracts. ^*^p < 0.05, ^**^p < 0.01 versus the positive control (NGF). (S) = sclerotium, (M) = mycelium.

The positive control (NGF) recorded 26.4 ± 3.6% of neurite-bearing cells (Figure 2). The extraction yield of extracts from the mushrooms are summarised in Table 2. The percentage of neurite bearing cells after treatment with aqueous extracts of *G. lucidum* (38.4 ± 4.2%), *L. rhinocerotis* (38.1 ± 2.6%), and ethanol extract of *C. militaris* (35.8 ± 3.4%) were significantly higher (*p* < 0.01) than NGF control by approximately 1.45-, 1.44- and 1.35-fold, respectively. Aqueous extracts of *G. frondosa* (33.7 ± 1.5%) and *P. giganteus* (33.4 ± 4.6%) were also shown to induce significantly (*p* < 0.05) higher neurite bearing cells compared to the NGF control. Meanwhile, the aqueous extracts of *L. rhinocerotis* mycelium, *H. erinaceus, G. neo- japonicum*, *P. pulmonarius*, as well as ethanol extracts of *H. erinaceus*, *P. pulmonarius* and *P. giganteus* showed varied neurite outgrowth stimulatory effects with average neurite bearing cells ranging from 26.4 ± 5.4% to 29.6 ± 2.2%. Further, these extracts showed no significant difference when compared to NGF control (*p* > 0.05). Among the plant extracts tested, wolfberry extract did not show any neurite outgrowth activity. The percentage of neurite bearing cells obtained after treatment with 20 μg/ml of ethanol extract of *G. biloba* (30.3 ± 2.5%) was better than curcumin which gave 26.4 ± 5.4% at 20 μg/ml. The five different mushroom extracts (*G. lucidum*, *L. rhinocerotis*, *P. giganteus, G. frondosa* and *C. militaris*) each at 20 μg/ml were selected for neurofilament staining.

**Table 2:** Extraction yield of aqueous and ethanol extracts from the studied mushrooms

**Mushrooms**	**Extract**	**Yield (%, w/w)**
*Lignosus rhinocerotis* (S)	Aqueous	3.56
*Lignosus rhinocerotis* (M)		4.65
*Hericium erinaceus*		8.56
*Ganoderma lucidum*		3.67
*Ganoderma neo-japonicum*		5.75
*Grifola frondosa*		3.54
*Pleurotus pulmonarius*		3.60
*Pleurotus giganteus*		6.70
*Hericium erinaceus*	Ethanol	4.50
*Cordyceps militaris*		3.45
*Pleurotus pulmonarius*		6.70
*Pleurotus giganteus*		5.30
*Lycium barbarum* (wolfberry)	Aqueous	9.76
*Gingko biloba*	Ethanol	n.d

(S) = sclerotium, (M) = mycelium. n.d. = not determined.

**Figure 3:**
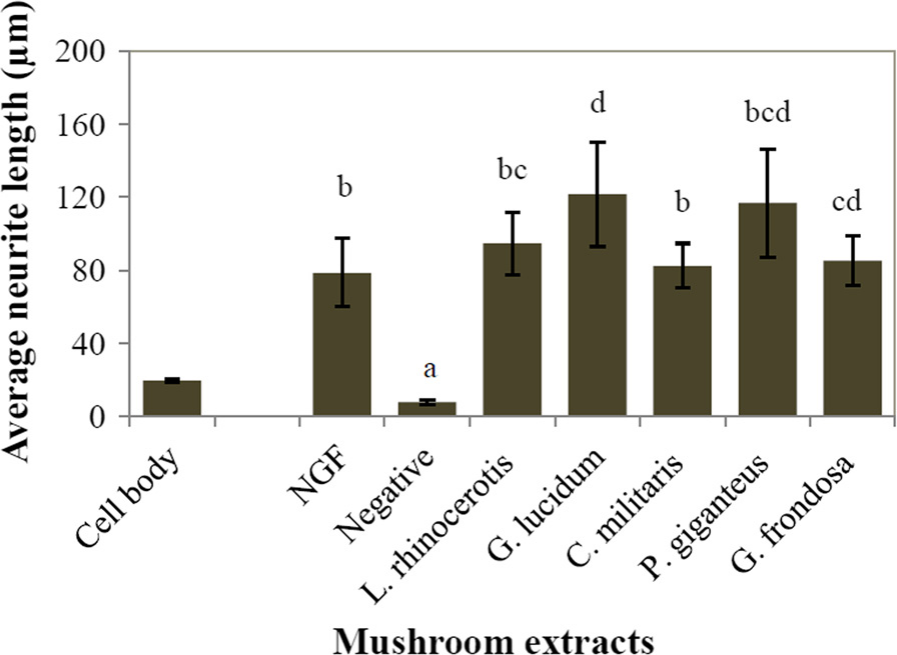
**The mean neurite length of N2a treated with different mushroom extracts at 20 μg/ml.** The results shown represent the mean ± SD; n = 3. Means not sharing a common letter were significantly different at *p* < 0.05. S = sclerotium, aq = aqueous, EtOH = ethanol.

**Figure 4:**
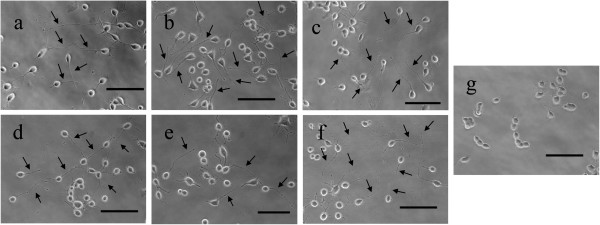
**Phase-contrast photomicrographs showing the effects of (a) NGF, (b) *G. lucidum*, (c) *L. rhinocerotis*, (d) *P. giganteus*, (e) *G. frondosa*, and (f) *C. militaris *on the morphology of differentiating N2a cells after 48 h. Untreated cells serve as control (g) and only contained 5% FBS as vehicle.** Arrows indicate typical neurites of N2a. Scale bar represents 20 μm. Photomicrographs of representative microscope fields were taken with a 20× objective.

The mean diameter of N2a cell body was found to be 19.45 ± 0.72 μm (Figure 3). To qualify as a “neurite”, the axon-like extension needs to be double or more than the cell body length of N2a. The average neurite length of NGF-stimulated cells was 78.58 ± 18.6 μm, which is approximately 4-time longer than the cell body. Cells treated with aqueous extract of *G. lucidum* were found to develop the longest mean neurite length i.e. 121.51 ± 28.6 μm (6.25-time longer than cell body), followed by aqueous extract of *P. giganteus* which recorded mean neurite length of 116.72 ± 29.5 μm (5.99-time longer than cell body). Figure 4 shows the morphology of differentiating N2a cells with neurites after 48 h of treatment with 50 ng/ml NGF (a) and 20 μg/ml of aqueous extracts of *G. lucidum* (b), *L. rhinocerotis* (c), *P. giganteus* (d) and *G. frondosa* (e); as well as ethanol extract of *C. militaris* (f).

### Immunofluorescence staining of neurofilament

**Figure 5:**
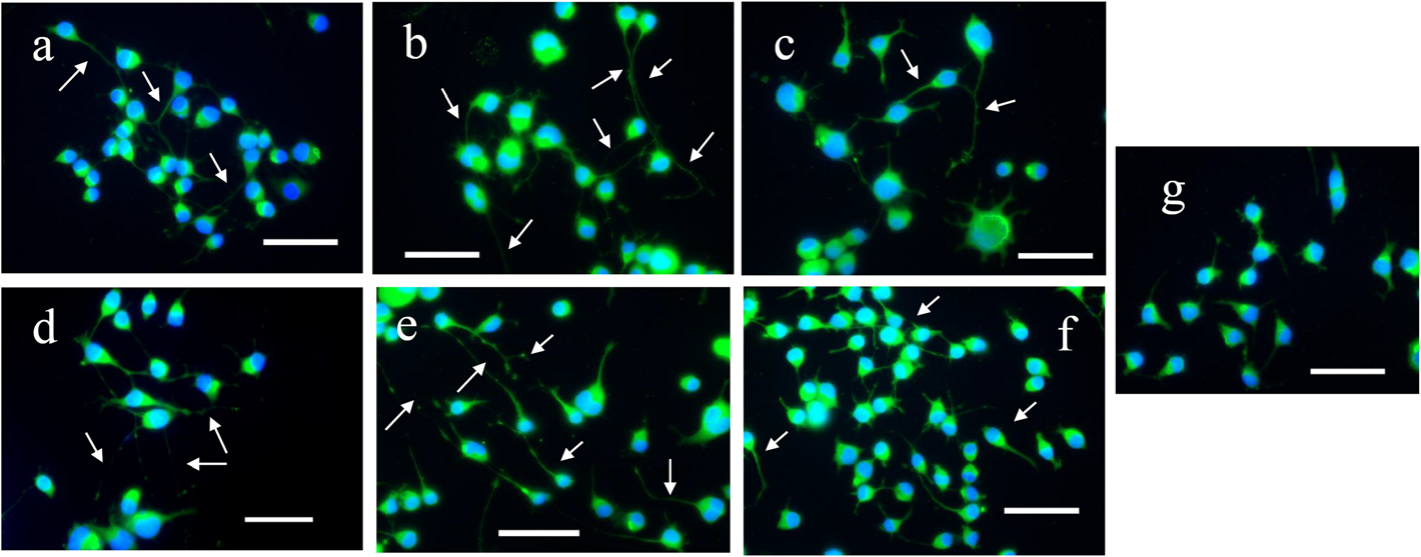
**Immunocytochemical staining of neurofilament in N2a cells treated with (a) NGF, (b) *G. lucidum*, (c) *L. rhinocerotis*, (d) *P. giganteus*, (e) *G. frondosa*, and (f) *C. militaris*, and untreated cells as control (g).** DAPI stains for nuclei blue, while anti-neurofilament 200 kD labeled with FITC stains neuronal cells green. Scale bar represents 20 μm. Arrows indicate neurite outgrowth. Photomicrographs of representative microscope fields were taken with a 20× objective.

Neurofilament belongs to a class of intermediate filament found in neuronal cells that provides specific support for axons. There is a direct relationship between neurite outgrowth and neurofilament expression as neurofilament protein levels increase with differentiation of cell lines [23]. Figure 5 shows the immunocytochemical labeling of neurons. The expression of neurofilament protein during neurite outgrowth was stained green while nuclei were stained blue.

### The cytotoxic effects of mushroom extracts on 3T3 and N2a by using MTT, NRU and LDH release assay

Table 3 shows the results of cytotoxicity screening of mushroom and plant extracts to N2a cells and 3T3 fibroblasts. The cytotoxicity determination are expressed as IC_50_ values, which is the concentration resulting in 50% inhibition of cell growth and proliferation after 24 h exposure. All the extracts of mushrooms and plants did not show cytotoxic effects to the two tested cell lines (IC_50_ ≥ 1 mg/ml in all cases). Three cytotoxicity endpoints were used in this experiment, which were MTT, NR uptake assay and LDH release assay. Interestingly, IC_50_ determined by NRU test and LDH test was higher than that of MTT. In general term, the ethanol extracts showed lower IC_50_ too.

**Table 3:** **IC**_**50 **_**values obtained by using different cytotoxicity assays- MTT, NRU and LDH release**

**Mushroom/Plant**	**Species**	**Extracts**	**Neuroblastoma 2a cells**			**3T3 embryonic fibroblast**		
			**IC**_**50 **_**(mg/ml) after 24 h**			**IC**_**50 **_**(mg/ml) after 24 h**		
			**MTT**	**NRU**	**LDH**	**MTT**	**NRU**	**LDH**
Culinary/medicinal mushroom	*Pleurotus giganteus*	Aqueous	4.07 ± 0.67^e^	6.95 ± 1.00^g^	-	2.16 ± 0.05^c^	2.67 ± 0.47^d^	-
	*Pleurotus pulmonarius*		2.85 ± 0.06^cd^	2.13 ± 0.87^cdef^	-	1.75 ± 0.14^bc^	2.60 ± 0.55^d^	-
	*Lignosus rhinocerotis* (sclerotium)		3.27 ± 0.77^d^	2.72 ± 0.65^ef^	-	5.63 ± 0.06^e^	5.93 ± 0.05^f^	-
	*Lignosus rhinocerotis*		2.43 ± 0.12^c^	1.75 ± 0.18^abc^	-	5.23 ± 0.17^e^	5.60 ± 0.1^f^	-
	(mycelium)							
	*Hericium erinaceus*		2.60 ± 0.52^cd^	2.85 ± 0.56^e^	-	3.43 ± 0.29^d^	3.53 ± 0.06^e^	-
	*Ganoderma lucidum*		1.35 ± 0.03^a^	1.52 ± 0.17^ab^	2.20 ± 0.52^ab^	1.19 ± 0.06^ab^	1.35 ± 0.02^ab^	1.50 ± 0.08^a^
	*Ganoderma neo-japonicum*		1.17 ± 0.006^a^	1.11 ± 0.03^a^	2.00 ± 0.53^ab^	1.47 ± 0.37^ab^	1.78 ± 0.56^bc^	1.58 ± 0.44^a^
	*Grifola frondosa*		2.72 ± 0.08^cd^	4.60 ± 1.10^f^	-	6.67 ± 0.87^f^	7.60 ± 0.1^g^	-
	*Pleurotus giganteus*	Ethanol	2.43 ± 0.10^c^	2.81 ± 0.15^e^	5.8 ± 0.10^c^	1.66 ± 0.56^bc^	1.96 ± 0.42^c^	1.73 ± 0.48^a^
	*Pleurotus pulmonarius*		2.30 ± 0.72^bc^	2.64 ± 0.16^def^	2.53 ± 0.96^bc^	1.27 ± 0.06^ab^	1.48 ± 0.05^abc^	1.67 ± 0.12^a^
	*Hericium erinaceus*		2.47 ± 0.50^c^	1.61 ± 0.51^ab^	2.10 ± 0.72^ab^	1.31 ± 0.03^ab^	1.84 ± 0.57^bc^	1.47 ± 0.31^a^
	*Cordyceps militaris*		1.65 ± 0.015^ab^	1.81 ± 0.10^abcd^	2.37 ± 0.21^b^	1.02 ± 0.08^a^	1.10 ± 0.02^a^	1.40 ± 0.29^a^
*Medicinal plant*	*Lycium barbarum*	Aqueous	4.37 ± 1.00^e^	6.96 ± 0.73^g^	-	8.40 ± 0.72^g^	7.86 ± 0.06^g^	-
	*Gingko biloba*	Ethanol	1.56 ± 0.08^ab^	1.66 ± 0.11^ab^	1.43 ± 0.39^a^	1.56 ± 0.58^abc^	1.37 ± 0.07^ab^	1.78 ± 0.07^a^
	Curcumin	Compound	1.13 ± 0.01^a^	1.15 ± 0.01^a^	1.30 ± 0.16^a^	1.16 ± 0.06^ab^	1.23 ± 0.03^ab^	1.60 ± 0.22^a^

The data represent the mean ± SD of three determinations. Means not sharing a common letter were significantly different at *p* < 0.05.

## Discussions

Eight species of medicinal mushrooms were investigated and categorised into two groups: culinary and non-culinary. The former group represents mushrooms that can be used for culinary purposes like preparing meal and cooking, especially from the fruiting bodies. This group (culinary-medicinal mushrooms) comprised of *P. giganteus*, *P. pulmonarius*, *H. erinaceus*, and *G. frondosa*. Medicinal mushrooms of no culinary properties are appreciated for their pharmacological merits and are not cooked as a meal. The fruiting bodies or sclerotia are often handpicked, ground to powder and subjected to various extraction methods before being used as a traditional medication [24]. *L. rhinocerotis, G. lucidum, G. neo-japonicum,* and *C. militaris* are non-culinary medicinal mushrooms.

*Ganoderma lucidum* (also known as Lingzhi in Chinese or Reishi in Japanese) has been widely investigated for its potential therapeutic benefits and longevity. Our results showed that aqueous extract of *G. lucidum* promoted neuritogenesis in N2a cells with 38.4 ± 4.2% of neurite bearing cells. This agrees with the finding of Cheung et al. [25] who showed that *Ganoderma* extract contained NGF-like compounds that mediated the neuronal differentiation and elongation of rat pheochromocytoma (PC12) cells. The *Ganoderma* neuroactive constituents that accounted for neurite outgrowth activity are triterpenoids, such as lucidenic acid [26], 7-oxo-ganoderic acid Z, ganolucidic acid A, methyl ganoderic acid A, ganoderic acid S1, and 4,4,14α-trimethyl-5α-chol-7,9(11)-dien-3-oxo-24-oic acid [27]. Further, the water-soluble polysaccharides of *G. lucidum* were shown to significantly (*p* < 0.05) reduce neuronal cell death and apoptosis of rat primary cortical neurons (model of brain cerebral ischemia) induced by oxygen/glucose deprivation treatment [19]. *Ganoderma* extracts may also provide mitigation to Parkinson’s disease as it was shown to prevent dopaminergic neuron degeneration by attenuating the pro-inflammatory response of microglial cells [28].

The tiger milk mushroom, *L. rhinocerotis* has been described as the national treasure of Malaysia as this macrofungus is rare and is often used as folk remedy to treat a variety of diseases. Consistent with our previous study [8], the sclerotia of *L. rhinocerotis* improved neurite outgrowth in N2a. Interestingly, in the present study, the sclerotial extract (38.4 ± 4.2% of neurite bearing cells) performed better than the mycelial extract (27.2 ± 2.9%). However, the active components in *L. rhinocerotis* that play the role in neurite outgrowth activity need further investigation. Lee et al. [15] highlighted that the protein or carbohydrate/protein complex of the cold water scerotial extract (4°C) is responsible for the antiproliferative activity against human breast and lung carcinoma. In this study, a hot water extraction approach was employed and we hypothesise that polysaccharides or triterpenoids rather than peptides are involved in the neurite outgrowth stimulatory activity of *L. rhinocerotis.*

Wild *Pleurotus giganteus* has been reported to be consumed by the indigenous tribes of Semai, Temuan, and Jakun in Malaysia [29]. This wild mushroom has been successfully domesticated for large scale production and is gaining popularity for culinary uses. The medicinal properties of *P. giganteus* are comparatively little as compared to *P. pulmonarius* (grey oyster mushroom). Recently *P. giganteus* (synonyms: *Lentinus giganteus* and *Panus giganteus*) was shown to have *in vivo* hepatoprotective effect in rat [13] and *in vitro* neuritogenic effects in PC12 cells [10]. On-going studies aimed at isolating and identifying the chemical constituents of the *P. giganteus* fruiting bodies indicate the presence of phenolics (caffeic acid and cinnamic acid), organic acid (succinic acid) and triterpenoids (unpublished data). The compounds may work synergistically and accounted for neuritogenesis.

*Cordyceps militaris* is a parasitic fungus that colonises moth larvae (Lepidoptera) and has been valued in Traditional Chinese Medicine for more than 2000 years. The major bioactive components in *C. militaris* include adenosine, cordycepin and polysaccharides [30]. Our finding is in agreement with the work by Lee et al. [21] where methanol extract of *C. militaris* was shown to significantly reverse the scopolamine-induced deficit in memory of rat and improve neurite outgrowth in N2a. Similar to our results, lysophosphatidylethanolamine isolated from *G. frondosa* (Maitake) was also reported to induce neuronal differentiation in PC12 cells, causing up-regulation of neurofilament M expression of PC12 cells [22].

It is well known that N2a cells, upon the withdrawal of serum, differentiate and elaborate neurites [31,32]. This well-defined neuronal model is often employed for studies relating to neuronal differentiation [33]. It is also a popular cell line in studying neurotoxicity as the brain is a first target in situations such as ageing and neurodegenerative diseases [34]. NGF is the most appropriate positive control in neurite outgrowth assays as its role in neural development have been characterised extensively as supported by Sofroniew et al. [35]. The therapeutic application of neurotrophins like nerve growth factor (NGF) is not possible as NGF cannot penetrate the blood–brain barrier. Studies indicate that lower-molecular-weight molecules may be a promising alternative for therapeutic intervention, for example, α-phenyl-N-tert-butylnitron [36]. However, most of the experiments testing natural products have been conducted *in vitro*, and few studies evaluated these compounds in the brain *in vivo*. Our previous study showed that aqueous extract of *P. giganteus* induced neuronal differentiation of PC12 cells *via* the activation of extracellular signal-regulated kinase (ERK) and phosphatidylinositol-3-kinase-Akt (PI3K/Akt) signaling pathways [10]. In fact, the most studied pathway controlling the consolidation of neurites involves signaling through neurotrophin receptors to a Ras-dependent, mitogen-activated protein kinase (MAPK) cascade [33]. At present, the precise neuritogenic signal transduction pathway involved in the actions of NGF, serum withdrawal, and the mushroom extracts is yet to be elucidated. It is anticipated that mushroom extracts (comprising of neuroactive polysaccharides or triterpenoids) under certain condition (serum deprivation) participate in triggering NGF signals, hence activating the downstream neuronal responses to axonal growth [37].

It is important that functional and health food remedies recommended for the prevention or treatment of diseases undergo safety assessment. Today, a number of natural products with potential biomedical application are being launched, although some could be potentially toxic when ingested at high doses or in combination with other medications [38]. Our results indicated that no cytotoxicity was detected for the concentrations tested. Notably, ethanol extract of *C. militaris* showed the lowest IC_50_ (*p* < 0.05) value against 3T3 fibroblast. Similarly, curcumin although was reported beneficial in neuroprotection [39], the IC_50_ value detected against N2a was the lowest by means of MTT, NRU and LDH assays. Elsewhere, *in vivo* toxicity evaluation of *Ganoderma boninense* (Pat.) was carried out [40] and a significant toxicity (IC_50_ = 640 μg/ml) against *Artemia salina* (brine shrimp) was demonstrated after 24 h. However, *Ganoderma* extract is granted safe on short-term exposure. Conversely, *in vivo* toxicity profiling of total triterpene fraction from *G. lucidum* against Swiss albino mice showed that ganoderma triterpenes did not possess significant toxicity [41] and administration of *G. lucidum* β–glucan (2000 mg/kg body weight/day) to Sprague Dawley rats did not cause toxicological abnormality [42]. Mutagenicity studies by means of *Salmonella typhimurium* also did not reveal any genotoxicity. Meanwhile, sub-acute toxicity study of the sclerotial powder of *L. rhinocerotis* by using rat model showed no treatment-related toxicity at 1000 mg/kg [43]. Taken together, beneficial mushroom extracts hardly exert any significant toxicity.

We have chosen more than one cytotoxicity assay namely MTT, NRU and LDH release assay to determine *in vitro* cell viability in order to increase the reliability of the results obtained and also to avoid over- or underestimation of the mushroom or plant toxicity. The mechanisms of the chosen assays are different. While MTT is based on the enzymatic conversion of MTT in the mitochondria, NRU assay is based on the dye uptake capability by lysosomes [44]. Both served as colorimetric assays, whereby viable and uninjured cells stain blue and red, for MTT and NRU assays, respectively. LDH release assay, on the other hand is based on the release of the enzyme into the culture medium after the disruption of cell membrane [45]. It is noteworthy that the toxicity profiles detected by the three different assays generally followed a similar trend although some results were not in agreement. For instance, no IC_50_ values were recorded for some mushroom extracts by using LDH assays, suggesting that LDH may be the least sensitive method among the three.

## Conclusions

The extracts of *G. lucidum*, *L. rhinocerotis*, *P. giganteus, G. frondosa* and *C. militaris* showed potential in promoting neurite outgrowth of differentiating N2a cells. The synergism of the various active entities in these mushroom extracts may be responsible for the neurite outgrowth activity and further experiments are warranted to isolate and identify the compounds. The signaling pathways involved is yet to be elucidated but based on our previous results, among other possibilities, phosphorylation and activation of the ERK and Akt may be involved. This study also showed the absence of embryotoxic and neurototoxic effects of the various mushroom extracts in 3T3 and N2a cells, respectively.

## Competing interest

The authors declare that they have no competing interests.

## Authors’ contributions

CWP carried out the study, performed the data collection, data management, statistical analysis, data interpretation, and manuscript writing. PD and VS conceived the study, participated in its design and coordination. MN participated in the design of the study. KHW took part in data interpretation. MN and VS contributed to conception of the design and execution of the study. All authors read and approved the final manuscript.

## Pre-publication history

The pre-publication history for this paper can be accessed here:

http://www.biomedcentral.com/1472-6882/13/261/prepub
